# Gut microbiota signatures in tissues of the colorectal polyp and normal colorectal mucosa, and faeces

**DOI:** 10.3389/fcimb.2022.1054808

**Published:** 2023-01-10

**Authors:** Xiaohui Zhong, Yuanyuan Wang, Jianmin Xu, Hong Cao, Feng Zhang, Xuesong Wang

**Affiliations:** ^1^ Wuxi School of Medicine, Jiangnan University, Wuxi, China; ^2^ Department of Hepatobiliary Surgery, Affiliated Hospital of West Anhui Health Vocational College, Liuan, China; ^3^ Department of Gastrointestinal Surgery, Affiliated Hospital of Jiangnan University, Wuxi, China; ^4^ Department of Endocrinology, Affiliated Hospital of Jiangnan University, Wuxi, China; ^5^ Department of Nutrition, Affiliated Hospital of Jiangnan University, Wuxi, China; ^6^ Department of Orthopedics, Affiliated Hospital of Jiangnan University, Wuxi, China

**Keywords:** colorectal polyps, gut microbiota, 16S rRNA, mucosa, *Fusobacterium*

## Abstract

**Background:**

Colorectal polyps are the most common precursors of colorectal cancer (CRC). The close relationship has been observed between colorectal polyps and gut microbiota. However, gut microbiota signatures among sampling sites in patients with colorectal polyps and healthy adults remain elusive.

**Aims:**

To learn about gut microbiota signatures in tissues of the colorectal polyp and normal colorectal mucosa, and faeces.

**Methods:**

We performed 16S rRNA gene sequencing and bioinformatic analysis for the microbiota in the normal colorectal mucosa, the colorectal polyps and faeces of adults with colorectal polyps (n = 24) and in faeces and normal mucosa of healthy adults (n = 16) in this preliminary trial.

**Results:**

The Ace and Chao indexes were higher in the normal colorectal mucosa and polyp tissues compared to faecal samples (*P* < 0.05). The composition of microbiota based on PCoA and ANOSIM analysis showed the significant differences only between faeces and tissues of the normal mucosa and polyp (*P* < 0.05). Based on the LEfSe analysis, the abundances of *Bacteroides*, *Prevotella-2* and *Agathobacter* were higher, whereas the abundances of *Haemophilus, Escherichia_Shigella*, *Fusobacterium* and *Streptococcus* were lower in faeces both in patients with colorectal polyp and healthy individuals, compared with those in the normal mucosa in two groups or polyp tissues. In healthy individuals, the abundance of *Fusobacterium* was significantly higher in the normal colorectal mucosa than in faeces. Moreover, there was no significant difference in the abundance of *Fusobacterium* between the normal colorectal mucosa and polyps in patients with colorectal polyps, but it was significantly higher in the mucosa and polyps than in faeces. Remarkably, the abundance of *Fusobacterium* in the normal colorectal mucosa was significantly higher in healthy individuals than in the polyp group.

**Conclusions:**

The microbial structure in faeces differs from that in tissues of polyp and normal mucusa. Additionally, *Fusobacterium* may be a normal colonizer in colonic mucosa, and an abnormal increase of *Fusobacterium* detected in faeces may be related with the injury of the colorectal mucosa. The difference of the faecal microbiota and mucosal microbiota should be carefully considered in studies on gut microbiota in patients with colorectal lesions.

## 1 Introduction

Colorectal polyps are protrusions on the surface of the colorectum, which are the most common precursors of colorectal cancer (CRC) (Sung et al., 2021). Colorectal cancer is mostly related to colorectal polyps because polyps are prone to transforming to a malignant carcinoma (Barberis et al., 2021). Modern medicine proves that the risk factors of colorectal polyps include ageing, male sex, high protein consumption (especially red meat), high-fat and low-fibre diet, smoking and excessive drinking (Zeller et al., 2014; Kordahi et al., 2021).

Studies have shown that there is a direct or indirect interaction between gut microbiota and intestinal diseases, such as inflammatory bowel disease, irritable bowel syndrome and CRC (Biondi et al., 2021; Cai et al., 2022). Our previous study found significant differences between mucosal and faecal microbiota in CRC patients, and the relative abundance of *Fusobacterium* was significantly higher in mucosa than in faeces (Li et al., 2022). Besides, a study confirmed that *Bacteroides fragilis* in faeces from patients with colorectal polyps can serve as a risk predictor for CRC (Kordahi et al., 2021).

Some studies have investigated the microbiota changes in the colorectal polyp microenvironment. For example, a study showed that *Fusobacterium mortiferum* increased in patients with intestinal adenomatous polyps (Liang S et al., 2020). In addition, compared with normal people, another study detected a higher number of *Fusobacterium nucleatum* in faecal samples from patients with adenomatous polyps (Rezasoltani et al., 2018). Besides, a study showed the regression of cap polyposis six months after oral administration of antibiotics (Okamoto et al., 2018). On the contrary, another study observed that the use of antibiotics increase the risk of colorectal polyps (Song et al., 2021). In general, the microbiota signatures among sampling sites in patients with colorectal polyps and healthy adults remain elusive.

This study analyzed the characteristics of faecal and mucosal microbiota in patients with colorectal polyps and healthy individuals by using 16S rRNA gene sequencing. In addition, the microbial signature of the colorectal polyp tissue and normal intestinal mucosal tissue was also compared. Overall, this study attempted to provide a reference for subsequent studies regarding gut microbial changes in the whole process of development from polyps to adenocarcinoma.

## 2 Materials and methods

### 2.1 Study population

Ethical approval was granted by the Ethics Committee of the Affifiliated Hospital of Jiangnan University, Wuxi, China (LS2022022), and this preliminary trial was registered at the Chinese Clinical Trial Registry (ChiCTR2200063806). All participants were 18–80 years old and were voluntarily enrolled prior to presenting for colonoscopy. Twenty-four patients with colorectal polyps were classified as the polyps group, while 16 healthy individuals were classified as the control group. The inclusion criteria including patients: 1. were diagnosed with proliferative polyps, inflammatory polyps and adenomatous polyps by colonoscopy and pathological results; Those patients: 1. with history of colonic cancer, colonic polyps or diabetes; 2. with use of antibiotics or probiotics in the past three months; 3. with symptoms of infection within 1 week; 4. with other intestinal diseases were excluded.

### 2.2 Sample collection

Faecal samples were self-collected by participants after enrollment and before bowel preparation. Colorectal polyps and normal mucosal biopsies were collected by the sterile biopsy forceps with colonoscopy following a bowel preparation. These tissues together with the mucus were collected, but the mucus was washed away with saline before DNA extraction. All specimens were labeled and immediately archived at -80°C (within 1 hour) until further processing. These specimens were labeled as the normal colorectal mucosal tissues (NC_), colorectal polyp tissues (CP_) and faecal sample (FS_) in the colorectal polyps group (_P) and the healthy control group (_C).

### 2.3 DNA extraction

DNA extraction was performed according to E.Z.N.A.^®^ Soil DNA Kit (Omega Bio-Tek, Norcross, GA, U.S.) instructions from manufacturer. 1% agarose gel electrophoresis was used to check the DNA integrity. The NanoDrop ND-1000 spectrophotometer (Thermo Fisher Scientifific, Waltham, MA) was used to measure the DNA concentration and purity.

### 2.4 16S rRNA amplicon sequencing

The variable region V3-V4 was amplified by an ABI Gene Amp^®^ 9,700 polymerase chain reaction thermocycler (ABI, CA, United States) using the following primer pair: 338F, 5’- ACTCCtacGGGagGCAGcagCAG-3’ and 806R, 5 ‘- GGACTACHVGGGTWTCTAAT - 3 ‘. Purified amplicons were pooled in equimolar and paired-end sequenced on the Illumina MiSeq PE300 platform (Illumina, San Diego, United States) according to the standard protocols by HonSunBio Technology Co. Ltd (Shanghai, China). The 16S rRNA sequencing was performed using a MiSeq Reagent Kit v3 (2 x 300, 600 cycles, Illumina Inc., San Diego, CA, USA). The OTU clustering of sequences based on 97% similarity was performed using UPARSE software (version 8.1) (Edgar, 2013), and individual sequences and chimeras were removed during clustering. The species classification of each sequence was annotated using the RDP Classifier and compared with the Silva database (SSU138), and the matching threshold was set to 70%.

### 2.5 Microbial analysis of sequences

After sequencing, the raw 16S rRNA gene sequencing reads were quality-fifiltered by fastp (version 0.21.0) (Chen et al., 2018) and merged by FLASH (version 1.2.12) (Magoc and Salzberg, 2011). The OTUs were clustered with a 97% similarity cutoff using UPARSE platform (version 8.1) (Edgar, 2013). Alpha-diversity focuses on the abundance and diversity of microbial communities, and commonly used diversity indexes include Ace, Chao, Shannon and Simpson indexes. Principal co-ordinates analysis (PCoA) and Anoism analysis were drawn based on Bray-Curtis distance matrix. The closer the distance, the closer the composition of the samples. Linear discriminant analysis effect size (LEfSe) was used for screening the key strains between groups (Segata et al., 2011). Besides, for revealing the potential differences in metabolism, phylogenetic investigation of communities by reconstruction of unobserved state analysis (PICRUSt2) (version 2-2.0.3-b) was employed for predicting the functional contents based on 16S rRNA gene data (Langille et al., 2013). The functional potential of the microbiota was predicted according to the guidelines with the rarefied OTU abundance table as the input. Relative predicted abundance of MetaCyc pathways was calculated by dividing the abundance of each pathway by the sum of all pathway abundances per sample. Relative contribution of each OTU to predicted pathways was calculated by dividing the contribution of each OTU by the sum of all contributions per sample.

### 2.6 Bioinformatic analysis together with statistical methods

Both the R software (version 4.1.2) (https://www.R-project.org) and QIIME (version 1.9.1) (Caporaso et al., 2010) were employed to analyze the sequencing data. The α-diversity indexes at OTU level, including richness estimator (Ace and Chao) and diversity index (Shannon and Simpson), were determined based on OTU table using QIIME. Spearman correlation analysis was performed between the relevant indicators and the corresponding gut microbiota, and the correlation coefficient was set as *R* > 0.4 or *R* < -0.4, and *P* < 0.05 were considered to be associated. The results of continuous variables were expressed as Mean ± standard error (Mean ± SEM), and categorical variables were expressed as frequency and percentage. All statistical analyses were performed by one-way ANOVA or t-test using GraphPad Prism software (version 9.0.0), and *P* < 0.05 represented statistically significant difference. Besides, “*” was marked in the corresponding plot for *P* < 0.05, and “**” was marked in the corresponding plot for *P* < 0.01.

## 3 Results

### 3.1 α-diversity among the microbiota in the normal colorectal mucosa, polyps and faeces

**Figure 1:**
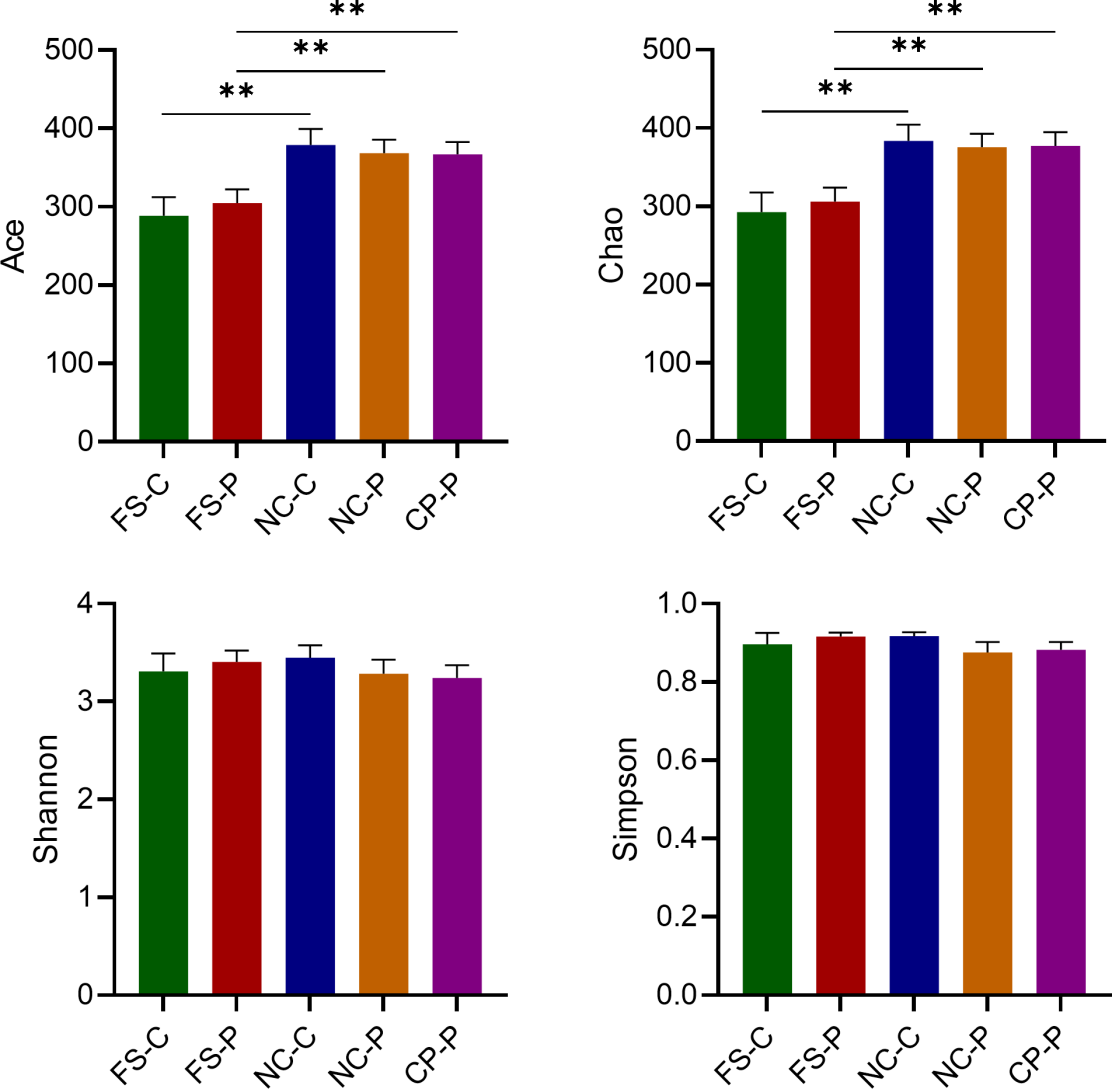
Analysis of α-diversity of microbiota in the normal colorectal mucosal tissues (NC_), colorectal polyp tissues (CP_) and faecal samples (FS_) in the colorectal polyps group (_P) and the healthy control group (_C). **P < 0.01.

As shown in **Figure 1**, the Ace and Chao indexes were higher in the normal colorectal mucosa and polyp tissues compared to fecal samples in patients with colorecctal polyps (*P* < 0.01). However, there was no significant difference in the Ace and Chao indexes between their normal colorectal mucosa and polyp tissues. In healthy individuals, the Ace and Chao indexes in the normal colorectal mucosa were higher than that in faeces (*P* < 0.05). Besides, the Shannon and Simpson index suggested that there was no difference in the community diversity among sampling sites both in patients with colorectal polyps and healthy individuals (*P* > 0.05).

### 3.2 Microbial communities in the normal colorectal mucosa, polyps and faeces

**Figure 2:**
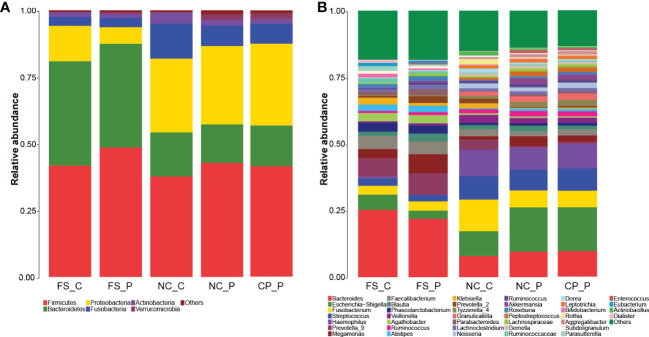
Microbial communities among sampling sites. **(A)** microbial communities at the levels of phylum; **(B)** microbial communities at the levels of genus.

The overall composition of faecal microbial communities appeared similar at the levels of phylum and genus in patients with colorectal polyps and healthy individuals, and so did the normal mucosal microbial communities. In patients with colorectal polyps, the composition of the microbial communities in colorectal polyps tissue appeared similar with that in the normal mucosa at the levels of phylum and genus. Nevertheless, microbial communities in faeces appeared different from that in tissues of the normal colorectal mucosa and polyp (**Figure 2**).

### 3.3 β-diversity among the microbiota in the normal colorectal mucosa, polyps and faeces

**Figure 3:**
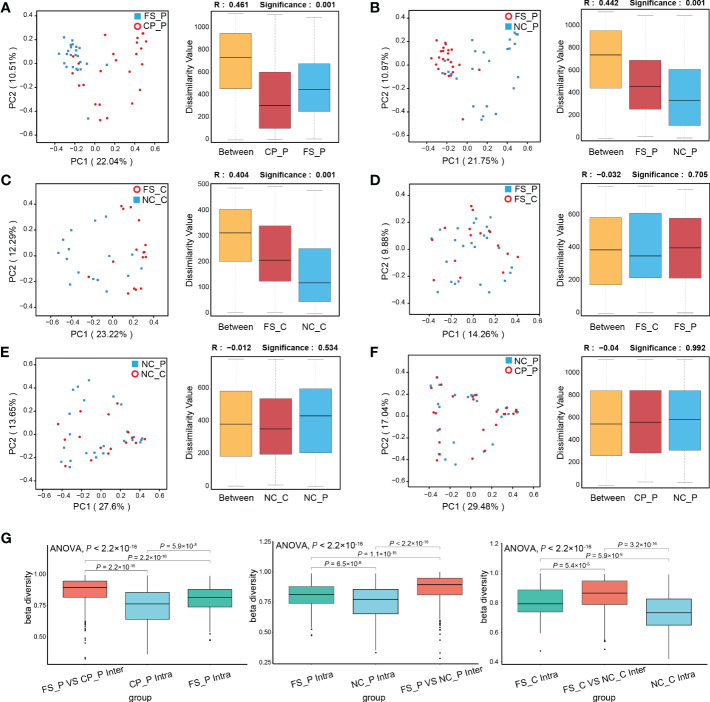
Analysis of β-diversity of microbiota among sampling sites. **(A-F)** PCoA and ANOSIM between groups; **(G)** the intra- and inter-individual Bray-Curtis distances using PCoA analysis.

As shown in **Figures 3A-C**, the structure of the faecal microbiota was significantly different from that of the microbiota in tissues of normal mucosa and colorectal polyp using PCoA based on Bray-Curtis distance (**Figures 3A-C**). Between healthy participants and patients with colorectal polyps, the structure of microbiota showed no difference in faeces, nor did in normal mucosa (**Figures 3D, E**). In addition, there is no difference in the composition of the microbiota in tissues of colorectal polyps and normal mucosa in patients with colorectal polyps (**Figure 3F**). Besides, the intra- and inter-individual Bray-Curtis distances were also evaluated at the OTU level using PCoA analysis. Actually, the inter-individual Bray-Curtis distance was significantly higher than the intra-individual distance among sampling sites in patients with colorectal polyps as well as in healthy individuals (**Figure 3G**).

### 3.4 Diffirential microbiota among sampling sites

**Figure 4:**
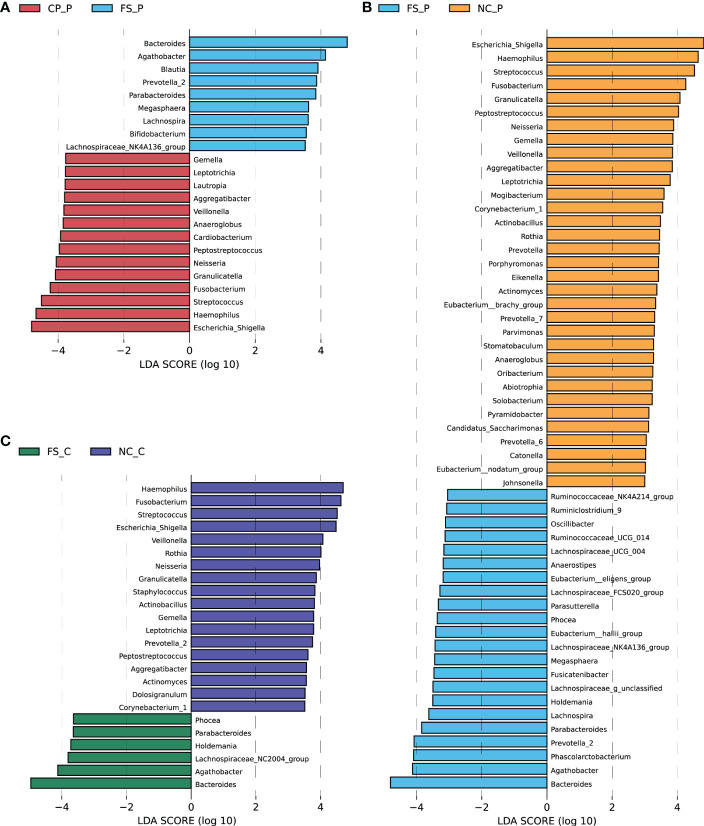
Differential abundant genera were screened out using LEfSe analysis **(A)** between colorectal polyps and faeces in the polyp group, **(B)** between normal mucosa and faeces in the polyp group, and **(C)** between normal mucosa and faeces in the control group.

Histograms of LDA scores (> 4.0) for the differential bacterial taxa in normal colorectal mucosa, polyp tissue and faecal samples were screened out using the LEfSe analysis. Compared with the faecal sample, in the polyp tissues, *Escherichia_Shigella, Haemophilus, Streptococcus, Fusobacterium* and *Granulicatella* were the dominant genera, while the abundance of *Bacteroides, Agathobacter, Phascolarctobacterium* and *Prevotella-2* were lower (**Figure 4A**). The differential genera in the normal colorectal mucosa and the faecal sample of patients with colorectal polyps were consistent with those between the faecal sample and the polyp tissue (**Figure 4B**). In the healthy participants, the abundance of *Haemophilus, Fusobacterium, Streptococcus, Escherichia_Shigella* and *Veillonella* were higher, while the abundance of *Bacteroides* and *Agathobacter* were lower in the normal colorectal mucosa compared with the faeecal sample (**Figure 4C**).

**Figure 5:**
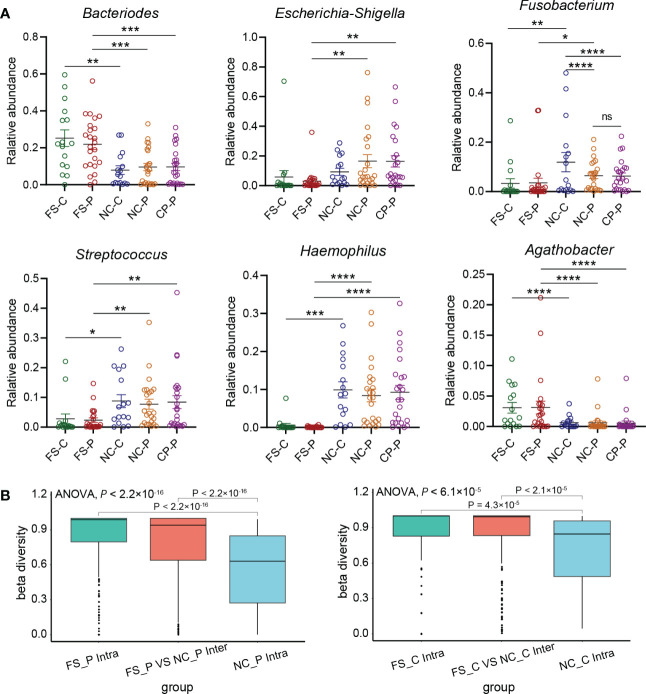
Key genera in five groups. **(A)** relative abundance of key genera in five groups selected by LEfSe including *Bacteroides, Escherichia_Shigella, Fusobacterium, Streptococcus, Haemophilus, Agathobacter*. *P < 0.05, **P < 0.01, ***P < 0.001, ****P < 0.0001. **(B)** the difference of *Fusobacterium* in inter-groups and intra-groups based on the Bray-Curtis distances using PCoA analysis.

We further compared genera in different sampling sites using Mann-Whitney statistic analysis. Consistent with the results of the LEfSe analysis, *Bacteroides*, *Prevotella-2* and *Agathobacter* were enriched in faeces, whereas *Haemophilus, Escherichia_Shigella*, *Fusobacterium* and *Streptococcus* were enriched in normal mucosa and polyps (**Figure 5A**). Remarkably, *Fusobacterium* was detected in the tissues of both colorectal polyps and the normal mucosa, and the relative abundance of *Fusobacterium* in the normal colorectal mucosa decreased in patients with colorectal polyps when compared to healthy subjects. Compared with faecal samples in healthy individuals, *Fusobacterium* was enriched in colorectal mucosa. Besides, when compared to faecal samples from patients with colorectal polyps, *Fusobacterium* was enriched in colorectal mucosa and polyps tissue. Moreover, the difference of *Fusobacterium* in inter-groups exceeds the difference in intra-groups based on the Bray-Curtis distances (**Figure 5B**). However, there was no significant difference in the abundance of *Fusobacterium* in their normal colorectal mucosa and polyp tissues. In addition, *Haemophilus* was rarely detected in faeces, while it was significantly enriched in tissues of colorectal mucosa and polyp.

Although there is no significance in the overall structure, some abundant genera were screened out using LEfSe analysis. As shown in **Supplementary Figure 1**, *Klebsiella* is more abundant in healthy individuals than in patients with colorectal polyps, both in faeces and in normal mucosa. The abundance of *Eubacterium_eligens* was higher, whereas the abundance of *Enterococcus* was lower in the normal mucosa of healthy individuals, compared with those of patients with colorectal polyps (**Supplementary Figure 1A**). Compared with healthy individuals, the abundance of *Butyricicoccus* in faeces was higher, whereas the abundances of *Lachnoclostridium* and *Erysipelatoclostridium* in faeces were lower in patients with colorectal polyps (**Supplementary Figure 1B**).

### 3.5 The functional differences among sampling sites

**Figure 6:**
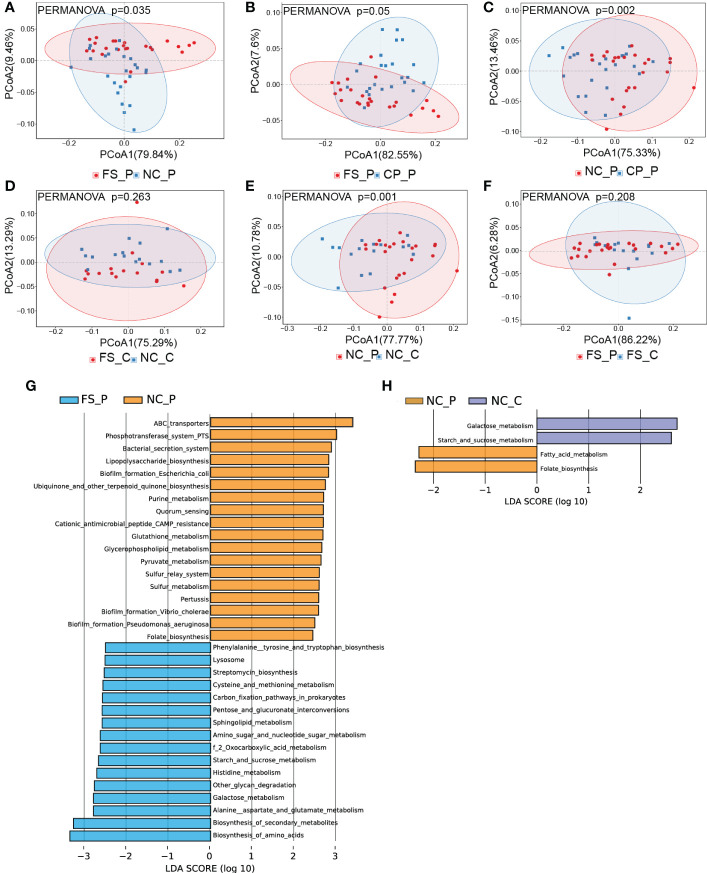
The overall functional differences in pathways among sampling sites. **(A-F)** the functional differences in pathways were displayed using PCoA analysis, and **(G, H)** the histograms of LDA scores (> 2.0) for the detailed differences in metabolic potential were displayed using LEfSe analysis.

For revealing the potential taxonomic differences corresponded to functional changes, we performed a predictive functional analysis using PICRUSt2 based on the 16S rRNA gene data. In patients with colorectal polyps, the metabolic pathways of microbiota in the normal mucosa were significantly different from those in faeces and polyp tissues, whereas those in faeces showed no difference from those in polyp tissues (**Figures 6A-C**). In healthy individuals, the metabolic pathways of microbiota in the normal mucosa were not different from those in faeces (**Figures 6D**). Significantly, the metabolic pathways of microbiota in the normal mucosa showed differences, while the metabolic pathways of microbiota in faeces showed no differences between healthy participants and patients with colorectal polyps (**Figures 6E, F**). In addition, compared with microbiota in faeces of patients with colorectal polyps, microbiota in the normal mucosa exhibits increased biofilm formation; and increased lipopolysaccharide, ubiquinone, and other terpenoid_quinone biosynthesis; and increased purine, glutathione, glycerophospholipid, and pyruvate metabolism (**Figure 6G**). By contrast, the microbiota in faeces is predicted to exhibit increased biosynthesis of amino_acids and increased alanine, aspartate, glutamate, glycan, histidine, starch, sucrose, cysteine, and methionine metabolism when compared with microbiota in normal mucosa of patients with colorectal polyps (**Figure 6G**). Besides, compared with microbiota in the normal mucosa of patients with colorectal polyps, the pathways of galactose, starch, and sucrose metabolism are predicted to increased, whereas the pathways of folate and fatty acid metabolism are predicted to decreased in those of healthy individual (**Figure 6H**).

## 4 Discussion

In this study, we found the differences in the microbial composition among different sampling sites from individuals with and without colorectal polyps. The β-diversity of the microbiota in the normal colorectal mucosa and polyp tissue are significantly different from that in faecal samples. Meanwhile, the metabolic pathways of microbiota in the normal mucosa showed differences, while those of microbiota in faeces showed no difference between healthy participants and patients with colorectal polyps, indicating there is a potential risk when we define colorectal mucosal microbiota with faecal microbiota. Microbial communities in the normal colorectal mucosa showed no difference between patients with colorectal polyps and healthy individuals, nor did in their faecal sample. However, it has been reported that the inter-individual differences in the mucosa-associated gastrointestinal microbiota were remarkable in healthy individuals (Kashiwagi et al., 2020). Collectively, the dysbiosis of fecal and mucosal microbiota was not remarkable in patients with colorectal polyps.

In this study, compared with faecal samples of healthy individuals, *Fusobacterium* was enriched in their normal colorectal mucosa. The relative abundance of *Fusobacterium* in the normal mucosa was higher in healthy individuals than in patients with colorectal polyps. Remarkably, we previously proved that *Fusobacterium* was extremely abundant in the tumor tissue, and gradually decreased in the normal colorectal mucosa and faeces (Li et al., 2022). Consequently, *Fusobacterium* was detected both in the lesion tissue and in the normal colorectal mucosa, which is consistent with the report that *Fusobacterium* can be detected in the intestinal mucosa of healthy individuals (Kashiwagi et al., 2020). In this study, the abundance of *Fusobacterium* did not increased in faeces when compared with that in the normal mucosa, which may be associated with the undamaged polyp tissue of the patients with colorectal polyps. Hence, there was no difference in the abundance of faecal *Fusobacterium* between healthy individuals and patients with colorectal polyps. Therefore, *Fusobacterium* may be the commensal colonizing bacterium in the normal colorectal mucosa. The abnormal increase of *Fusobacterium* detected in faeces may be associated with the injury of the colorectal mucosa.

Besides, it has been reported that *Klebsiella* spp. are opportunistic pathogens which are normally found in gut microbiota of healthy individuals (Dong et al., 2022). Studies have proved that *Eubacterium_eligens* and *Butyricicoccus* strongly promote the production of the anti-inflammatory cytokine (Eeckhaut et al., 2013; Chung et al., 2017). The genus *Enterococcus* and *Erysipelatoclostridium* are of great relevance to human diseases for their role as major causative agents of severe infections (García-Solache and Rice, 2019; Zakham et al., 2019). A study revealed a novel faecal *Lachnoclostridium* marker for the non-invasive diagnosis of colorectal adenoma and cancer (Liang J et al., 2020). In this study, *Enterococcus, Lachnoclostridium* and *Erysipelatoclostridium* are potentially to be causes of aggravating the condition in patients with colorectal polyps. Although there is no significance in the overall structure, these abundant genera could be explored as potential target for the treatment of infection.

In this study, the richness of gut microbiota in the normal colorectal mucosa and polyps was significantly higher than in faeces, which was inconsistent with the results of some studies (Pop et al., 2020; Zhou et al., 2021). Accumulating studies have explored the gut microbiota in individuals and identified a wide range of different bacterial groups associated with carcinogenesis, including *Bacteroides, Fusobacterium*, *Escherichia and Streptococcus* (Mangifesta et al., 2018). An abnormal regulation of TLRs in relation to gut microbial quantity may contribute to carcinogenesis. TLR2 and TLR4 expression was directly associated with the *Fusobacterium, Enterococcus and Streptococcus* (Rezasoltani et al., 2020). One study showed that *Enterococcus* release enterotoxins and reactive oxygen contributing to DNA damage, inflammation, and injury to the epithelial barrier (Pop et al., 2020). In contrast, *Agathobacter*, mainly in faeces, positively correlates with the outcome of patients with CRC (Martini et al., 2022). Besides, *Sutterella* and *Escherichia_ Shigella* being the most representative genera, appeared to be associated with malignancy (Mori et al., 2018). Nevertheless, in this study, *Escherichia_Shigella* was not detected abundant in faeces but in the polyp and the mucosal sample. These discrepancies showed that the dysbiosis was more severe in colorectal mucosa than in faeces. Notably, the overall hospital mortality of 28.1% was observed among patients with bacteremia due to *Haemophilus* and *Aggregatibacter* species (Chien et al., 2021). However, *Haemophilus* is rarely detected in faeces in this study, while it is significantly enriched in normal colorectal mucosa and polyps. Therefore, it may indicate the damaged colorectal mucosa once the *Haemophilus* is detected with high abundance in faeces.

Overall, faeces were usually adopted as the sample for the convenience of sample collection in studies on gut microbiota. However, the colorectal faecal microbiota is a mixture of bacteria in the intestinal lumen and drops from mucosa. The differential taxa in faeces between healthy individuals and patients with colorectal polyps are not the same as those in colorectal mucosa (Eckburg et al., 2005). Besides, it has been reported that the biofilm plays a protective role in adherent microbiota (Baumgartner et al., 2021). In this study, the microbiota in faeces is predicted to exhibit increased nutrient metabolism when compared with microbiota in the normal mucosa of patients with colorectal polyps. On the contrary, the microbiota in the normal mucosa is predicted to exhibit increased biofilm formation, which helps to avoid environmental influences and to protect mucosal bacterium. It has been found that the mucosal microbiota is superior to faecal bacteria in distinguishing disease phenotypes (Čipčić Paljetak et al., 2022), which was also proved by this study. Since bacteria in close contact with the epithelium may have greater potential to impact the progression of colorectal pathological changes (DeDecker et al., 2021). Therefore, this study provides a perspective on the unrepresentative role of faecal microbiota for mucosal microbiota.

Nevertheless, some limitations existed in this study. Although the process of library preparation and DNA sequencing are identical for faeces and tissues, a few differences in DNA extraction may lead to bias in the results. Another limitation was the number of cases enrolled. In addition, gut microbiota in patients with colorectal polyps is not significantly different from that in healthy individuals. The dramatically change of gut microbiota can be provoked in colorectal carcinogenesis rather than the formation of colorectal polyps.

## 5 Conclusions

This study suggests that the richness of the colorectal polyps and normal mucosal microbiota were significantly higher than that of faecal microbiota. Significantly, *Fusobacterium* may be the normal colonizing bacteria of colonic mucosa, and an abnormal increase of *Fusobacterium* detected in faeces may be related with the injury of the colorectal mucosa. Meanwhile, the signature of mucosal microbiota cannot be replaced by the faecal microbiota, and both should be carefully considered in studies of gut microbiota in patients with colorectal lesions. Nonetheless, during the development of colorectal polyps, the contribution of faecal and mucosal microbiota remains to be investigated.

## Data availability statement

The data presented in the study are deposited in the NCBI repository, accession number PRJNA888242. The data has been released.

## Ethics statement

This study was approved by the Ethics Committee of the Affifiliated Hospital of Jiangnan University, Wuxi, China (LS2022022). The trial has been registered at the Chinese Clinical Trial Registry (ChiCTR2200063806). Written and verbal informed consent was obtained from all subjects.

## Author contributions

YW, JX and FZ designed the trial. YW, JX and XZ conducted the study. XZ and FZ analyzed and interpreted the data. XW, FZ and HC were responsible for project administration and supervision. XZ wrote the first draft of the manuscript and had primary responsibility for the manuscript’s final content. All authors critically revised the manuscript. All authors contributed to the article and approved the submitted version.

## Supplementary material

Supplementary Figure 1 Differential abundant genera were screened out using LEfSe analysis between healthy individuals and patients with colorectal polyps. **(A)** the genera in the normal colorectal mucosa, **(B)** the genera in faeces.
