# Supplementary figures and images for: Gut microbiota signatures in tissues of the colorectal polyp and normal colorectal mucosa, and faeces
Source: Front Cell Infect Microbiol. 2023 Jan 10;12:1054808. doi: 10.3389/fcimb.2022.1054808 (PMC9871776; doi:10.3389/fcimb.2022.1054808)

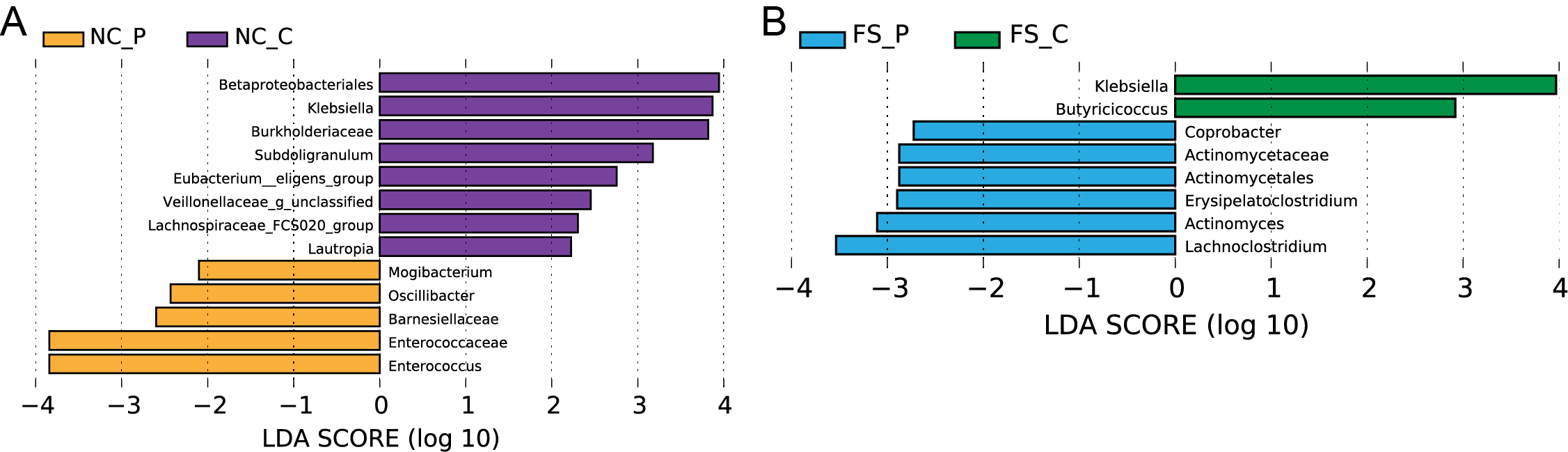

Supplement: Supplementary Figure 1 — Differential abundant genera were screened out using LEfSe analysis between healthy individuals and patients with colorectal polyps. (A) the genera in the normal colorectal mucosa, (B) the genera in faeces. [file Image_1.tif]
